# Supplementary material for: The complex evolution and genomic dynamics of mating-type loci in Cryptococcus and Kwoniella
Source: PLoS Biol. 2025 Oct 3;23(10):e3003417. doi: 10.1371/journal.pbio.3003417 (PMC12510652; doi:10.1371/journal.pbio.3003417)
Supplement: Attachment — Submitted filename: Response_to_reviewers.pdf [file pbio.3003417.s022.pdf]

Response to Reviewers (PBIOLOGY-D-25-00903R1)

Reviewer #1's comments:

Coelho et al. present an extensive analysis of a large number of genomes from various strains and species of Cryptococcus and Kwoniella fungi, focusing on the mating type (MAT) regions and chromosomes. The study contributes a valuable resource by providing numerous telomere-to-telomere assemblies. While many of the hypotheses explored—such as transitions from tetrapolar to bipolar mating systems, introgression events, and mating type locus rearrangements—have been analyzed before they have not previously been studied in such depth or with such a broad comparative framework. The authors extend the analyses, comparing MAT differences between Kwoniella and Cryptococcus, assess genetic variation within and between these genera. Given that Cryptococcus includes important human pathogens, a detailed understanding of its mating system evolution has clinical as well as evolutionary significance.

The manuscript covers a wide range of topics: introgression of the MATa locus between Cryptococcus lineages, reorganization of the a1 and a2 alleles in Kwoniella, chromosomal fusions and centromere loss, duplications of mating-type pheromones, and gene gains and losses. The strength of the study lies in its comprehensive analysis of MAT loci across these species. However, this breadth also emerges as a weakness: despite the exceptional quality of data and analyses, the overarching narrative of the paper remains unclear. The manuscript attempts to cover too much ground, lacks a unifying hypothesis, and is overly verbose. It reads more like three separate projects—one per genus, plus a comparative component—condensed into a single paper. I will first address this issue, followed by specific comments on the content.

I believe the manuscript would benefit substantially from a restructured organization of topics and a more concise presentation of results.

At present, the flow jumps between different levels of analysis: from overall MAT structure and breeding systems, to P/R in Kwoniella, then to Cryptococcus P/R, back to general MAT structure in Cryptococcus, followed by mating-type chromosome fusions, then back again to Kwoniella chromosomal changes and breeding systems. A more thematic structure—e.g., a main section on "Fusion Events" comparing the two genera, with genus-specific findings as subheadings—would enhance coherence and focus. Figure 10 seems intended to synthesize the major findings, but this integrative perspective is not well reflected throughout the manuscript.

Response: We thank the reviewer for their thoughtful assessment of our manuscript. We appreciate the concern, shared by three reviewers, that the manuscript is long. However, while it covers a wide range of topics, reflecting the diversity of evolutionary processes acting on the *MAT* loci across *Cryptococcus* and *Kwoniella*, we feel these aspects are interrelated. Our intention was to present a comprehensive comparative framework, as the availability of numerous telomere-to-telomere assemblies provided a unique opportunity to address and revisit different questions in parallel. We think that dispersing these analyses across multiple manuscripts would fragment the overall narrative and weaken the integrative perspective that we aim to provide.

That said, we acknowledge the reviewer's concern that the breadth of topics may make the narrative seem diffuse. In revising the manuscript, we have streamlined overly verbose passages while condensing or moving some more technical or detailed aspects to S1 and S2 Texts. To further

aid readability, we also introduced subheadings within the Results and Discussion sections, so that related topics (e.g., *P/R* locus expansions, and fusion events) are more clearly delineated. We note that it has at times been challenging to reconcile the need for conciseness with simultaneous requests from reviewers for expanded discussion in specific areas. We have aimed to balance these perspectives by simplifying where possible while ensuring that key points raised by the reviewers are still fully addressed.

Below, we address each of your additional comments and outline the revisions made to our manuscript accordingly. Please note that line numbers refer to the revised clean manuscript without tracked changes.

In addition to the structural issues, the writing style contributes to the lack of clarity. The manuscript adopts a storytelling approach that is overly descriptive and often loses sight of the central message. For instance, the section discussing the introgression of the mata locus (lines 409-432) initially suggests that introgression may have occurred, then dismisses this possibility due to structural rearrangements, only to return to the introgression hypothesis based on SNP data. Presenting the conclusion first, followed by supporting evidence, would improve clarity and focus. This issue is also apparent in the introduction: it is lengthy, and only in the final paragraph does it become clear what the paper aims to address. More broadly, the text is excessively wordy, and the authors could significantly reduce its length without sacrificing content or depth.

Response: We thank the reviewer for the feedback on writing style and clarity. Again, we recognize that parts of the manuscript are descriptive given the detailed nature of the data and analysis, but this reflected an effort to also provide sufficient detail for readers particularly interested in the evolutionary genetics of mating systems in fungi. At the same time, we agree that clarity of the central message is paramount, and in revising we sought to balance accessibility for the general reader with sufficient detail for specialists.

Regarding the specific point on the introgression of the *MAT***a** locus, we clarified the logic to avoid any impression that we dismissed the introgression hypothesis. We now explicitly state that "*This structural divergence did not contradict the introgression model but raised questions about how such differences could be reconciled with an introgressed origin*" (**lines 377-378**). This revised framing makes clear from the outset that structural divergence complicated but did not refute the introgression scenario proposed by Desjardins et al. (2017; PMID: 28611159), thereby setting up the rationale for our SNP-based reanalysis using the higher quality assemblies generated in this study.

For the introduction, we appreciate the observation that the overarching aim becomes clearest in the final paragraph. We think this follows a common structure in biology manuscripts, where context builds toward a concise statement of aims. Nonetheless, we have revised the introduction to trim overly descriptive passages.

Finally, we acknowledge the broader point about wordiness. We reduced text length where possible, but also addressed other reviewers' requests for expanded discussion of particular topics. We have aimed to reconcile these perspectives by simplifying where possible, while retaining the detail needed to address specific comments and to present a comprehensive comparative framework.

Comments on content:

Difference between Kwoniella and Cryptococcus:
The authors have two genera that evolved differently, but with convergence in certain aspects. Currently, the manuscript mostly describes these independently, but the comparison of why certain things might have occurred differently are not properly discussed. What are the main ecological differences? What is the population structure of each genus? Why are pheromones expanding in one, but not in the other? It would be interesting if hypotheses are given and tested.

Response: We thank the reviewer for highlighting the need for a more explicit comparative framing between *Cryptococcus* and *Kwoniella*. At present, population structure has been investigated mainly in pathogenic *Cryptococcus* species, whereas for non-pathogenic *Cryptococcus* and for *Kwoniella* it remains virtually unknown. A major limitation is that most species are known from only single or very few isolates, which prevents meaningful assessment of their natural population dynamics or ecological associations, unlike in fungal models such as plant parasites that typically have well-characterized host-specific populations. We discuss these challenges in the revised Discussion (**lines 770-784**).

We have also added hypotheses to address why pheromone gene duplications recur in *Cryptococcus* but not in *Kwoniella* (**lines 623-627**). While the lack of ecological and population-level data prevents formal testing, we now emphasize a plausible ecological distinction: *Kwoniella* species produce spores embedded in hyphal tissue, likely relying less on pheromone-mediated long-range signaling, whereas *Cryptococcus* species form aerial spore chains where efficient signaling over distance could be advantageous. This difference in dispersal biology may help explain why repeated pheromone gene duplications are observed in *Cryptococcus* but absent from *Kwoniella*. Of course this remains to be formally tested.

Mating type content:
What is the importance of non-MAT genes in the mating type? The authors mention that in some species there is extension and incorporation of novel genes for cell organelle inheritance for example, however it is unclear why this would happen in general. In isogamous species, the mating types function is mostly limited to regulation of conjugation and a ploidy level physiological switch. Neutral hypotheses are generally not discussed by the authors.

Forces leading to expansion of the regions of reduced recombination are highly debated running from neutral, to beneficial for dosage compensation or heterozygosity (see for example recent papers 10.1016/j.tig.2024.03.005, 10.1126/science.abj1813, 10.1093/evolut/qpae060, 10.1093/evolut/qpad023). The authors tend to imply that gene incorporations are adaptive, however a neutral hypothesis should not be dismissed. For example Line 269: "has expanded to include two additional genes [..] (also present in the MAT locus of Cryptococcus pathogens". This sentence is ambiguous or even misleading. Without giving the synteny of the outgroup, it is unclear if these have been recruited, as is suggested by 'expanded', and 'also present' suggests even that this would be adaptive as it happened twice. I might have missed something, but to me it seems more parsimonious that these genes were lost in the other clades which means they are not adaptive.

Response: We thank the reviewer for these insightful comments. We agree that the forces driving the expansion of recombination-suppressed regions such as *MAT* are debated and may include both adaptive and neutral processes. To avoid implying selective recruitment, we have revised the manuscript to use more neutral language (e.g., “entrapped,” “retained,” or “included”) rather than

phrasing such as “expanded to include,” which could suggest adaptive gain (see e.g. **line 593**). In this context, we now clarify that the incorporation of non-*MAT* genes into *MAT* loci is most likely explained by neutral structural processes, particularly inversions or other rearrangements, that entrapped neighboring genes into recombination-suppressed regions (**lines 593-596**) .

This interpretation applies to cases such as *BSP3*, which is found within the *Kwoniella* clade G P/R locus as well as in the *Cryptococcus MAT* locus. Rather than repeated adaptive recruitment, a more parsimonious explanation is independent entrapment facilitated by similar ancestral localization near the locus. Indeed, as shown in S5 Fig, *BSP3* was likely relocated into the clade G *P/R* locus by an inversion (INV3), distinct from the proposed rearrangements shaping clades E/F. The presence in both lineages is more consistent with convergent neutral processes than with adaptive parallelism.

More broadly, we note that some *MAT*-linked genes (*STE20*, *SPO14*) are primarily associated with post-mating development, others (*DBP10*, *REI1*, *NDC80*) appear unrelated to sexual reproduction. Their inclusion within *MAT* loci is also likely explained by neutral entrapment rather than adaptive recruitment. While alternative scenarios such as gene co-regulation with *MAT* genes or antagonistic selection (i.e., different alleles being optimal in the two mating types) are conceptually interesting, the current data do not allow these to be tested, and for reasons of scope we have not elaborated on them in this manuscript. However, we now cite two recent reviews (**line 596**) emphasizing the role of neutral processes in the expansion of recombination-suppressed regions (Hartmann et al. 2021, PMID: 33113229; Jay et al. 2024, PMID: 38677904).

Whatever the cause of mating type region expansions, its consequences will be degeneration of genes caught in these regions. To overcome pseudogenization transfer of genes might occur to overcome this degeneration. With the extensive data at hand, including ancestral reconstructions, the authors might be able to analyze this specific aspect of mating type content. For example, as mentioned but not extensively analyzed, fusions of the mating types likely occur for increased sib-sib compatibility but as a consequence will trap many non-mating type related genes in the mating types. Fast reorganization of the mating types might be driven to overcome this degeneration.

In line with this, the very interesting finding that introgression occurred of the mata in Cryptococcus lineages (line 418-) could be associated with degeneration. I suggest to include a reference to Corcoran et al. (10.1101/gr.197244.115), who suggested that introgression of mating types might occur to revive mating type function which due to Muller's ratchet is prone to degenerate. According to their hypothesis introgression might be a means to rejuvenate the genome.

Response: We thank the reviewer for raising this stimulating point. We agree that suppressed recombination can accelerate degeneration of *MAT*-linked genes, and that structural reorganization might, in principle, act to “rescue” them. Such a process would require parallel movement in both mating types to restore recombination, making it less straightforward than introgression, which may represent a more efficient mechanism for “rejuvenating” *MAT* alleles.

One example consistent with relocation has been reported in *C. deneoformans* (Fraser et al. 2004, PMID: 15538538): the genes *BSP3* and *IKS1*, which reside within *MAT* in *C. neoformans* and the *C. gattii* complex, are instead positioned immediately flanking the *MAT* locus in *C. deneoformans*. Their similar sequence between the two mating types suggests they were “evicted” from *MAT*, showing that reorganization can in principle relocate genes out of recombination-suppressed regions.

We also note that some of the genes now embedded within *MAT* or *P/R* loci are confirmed essential (e.g., in *C. neoformans*; Bian et al. 2024, PMID: 39677606), which likely constrains their evolutionary trajectory. For such genes, pseudogenization is unlikely to be tolerated, so their persistence within recombination-suppressed regions is expected even without “rescue” by reorganization or introgression. Gene conversion within *MAT* during a–α sexual reproduction can also counteract degeneration by repairing deleterious mutations using the opposite allele as a template, thereby slowing divergence between alleles. Thus, the evolutionary fate of *MAT*-linked genes likely reflects a balance between constraint (for essential genes) and degeneration or relocation (for nonessential ones).

That said, a systematic analysis of the frequency and implications of such events is not straightforward, given the extent of intraspecific variation observed even within *C. neoformans*. For this reason, we did not develop a dedicated section on this hypothesis in the present manuscript, but we fully agree it represents an important direction for future study and we hope the reviewer concurs.

Finally, we have added a citation to Corcoran et al. (2016, *Genome Res.*, PMID: 26893460) when discussing introgression in *C. neoformans* (**lines 642-645**), which now states:

*“Introgression events, such as the transfer of MAT***a** *alleles between C. neoformans lineages, may further buffer against degeneration by resetting mutational load [128], thereby helping maintain complex MAT configurations.”*

<u>Mating type locus fusions:</u>
About the term 'pseudobipolar': Is it possible to test if the linkage of the two mating types loci is expected based on a neutral scenario, due to the repeated fission and fusion of chromosomes? Or rather that this was likely driven by selection. If this was the only fusion, the chance these two chromosomes merged was 1/(14*13), which is rather small, but if many occur, this could be due to random chance. Even though the distance between the loci is large, it could be that the two loci are linked nevertheless, as recombination can be suppressed over large distances as shown in Neurospora tetrasperma or Microbotryum (10.1038/s41467-017-01317-6, 10.1111/nph.17039).

Response: We thank the reviewer for this thoughtful comment on the evolutionary dynamics of *MAT* locus linkage and the use of the term “pseudobipolar”. This raises two important and related questions: (i) whether recombination suppression might result in functional linkage even across large physical distances, and (ii) whether such linkage is more likely to arise by chance or via selection.

Regarding the first point, the pseudobipolar configuration (defined as the presence of both *P/R* and *HD* loci on the same chromosome but separated by a large distance) is currently observed only in *K. europaea*. In this species, the two loci are separated by ~8.5 Mb. Based on this distance, we consider it unlikely that the loci are genetically linked. However, we acknowledge that recombination suppression can span megabase-scale regions in some fungi, such as in the true bipolar systems of *N. tetrasperma* and *Microbotryum*. Thus, we cannot presently exclude the possibility that recombination is suppressed in *K. europaea*, and this configuration could represent either a transient intermediate state or an early step toward functional linkage. Importantly, if recombination between the loci does occur, the system would remain functionally tetrapolar, suggesting this fusion could instead represent a stochastic rearrangement with limited evolutionary consequence. Unfortunately, *K. europaea* is not known to undergo sexual reproduction in laboratory crosses

(Guerreiro et al., 2013; PMID: 23524993), which prevents direct assessment of recombination between the two *MAT* loci.

Regarding the second point, we agree that under a 14-chromosome ancestral state, the probability of a single, specific fusion between the *P/R*- and *HD*-bearing chromosomes is low. Our previous work (Coelho et al., 2024) also showed that *Kwoniella* species in clade E experienced extensive chromosomal fusions, reducing chromosome number from 14 to just 3. Despite this genome compaction, three of the four species in the clade (*K. mangrovensis*, *K. botswanensis*, and *Kwoniella* sp. B9012) retain a tetrapolar configuration, with the *P/R* and *HD* loci remaining on separate chromosomes. Only *K. europaea* exhibits the pseudobipolar arrangement, which appears to result from a translocation between ancestral chromosomes 1 and 2. Thus, even in a lineage with rampant karyotype reshuffling, *MAT* locus co-location has occurred only once. This suggests that such configurations may represent rare events that, depending on their impact on recombination and mating success, could either be selectively retained or remain transient and effectively neutral in the absence of functional linkage.

We have clarified these points in the revised manuscript (**lines 740-749**) and thank the reviewer for prompting this deeper discussion.

Breeding system:

It is very exciting to see experimental test of the potential for sexual reproduction. It would be great if there was more known on mating in naturel. Mating types do indicate the potential for haploid selfing or outcrossing, the natural occurrence of outcrossing and inbreeding are not assessed. The authors write: "While genomic analysis of MAT loci content and organization allows for inferences about potential breeding systems (heterothallism vs. homothallism), experimental mating assays remain the most definitive approach for confirming sexual reproduction and viable progeny production." I disagree. The potential for outcrossing, does not indicate the occurrence of outcrossing. Heterothallic species are compatible with a quarter (tetrapolar) or half (bipolar) of their sibs and thus inbreeding can be rife in heterothallics. A better understanding can be obtained by assessing heterozygosity and runs of homozygosity (10.1111/jeb.13780) in aneuploid or diploid natural isolates (when available) or by studying LD decay which is also possible in haploids (10.1073/pnas.1503159112). This will give information not only on whether sexual reproduction is possible, but also actually occurs in nature.

Response: We thank the reviewer for this thoughtful comment and agree that confirming the potential for mating does not imply that outcrossing occurs in nature. Our original intention in stating that "*experimental mating assays remain the most definitive approach for confirming sexual reproduction and viable progeny production*" was to emphasize that such assays are an established approach to determine whether a species is capable of completing a sexual cycle under controlled laboratory conditions, not to infer mating patterns in natural populations. We did not mean to suggest that the presence of compatible mating types or laboratory-confirmed mating capacity implies natural outcrossing.

Additionally, we fully agree that assessing linkage disequilibrium (LD) decay from population-level genomic data offers a more direct means to evaluate whether mating, and particularly outcrossing, occurs in nature, or whether inbreeding predominates. As the reviewer rightly notes, measures of heterozygosity and runs of homozygosity are less informative in these cases, as the vast majority of *Cryptococcus* and *Kwoniella* isolates are haploid. While LD-based approaches are extremely valuable, we have now cited the references suggested by the reviewer and clarified our wording in

the Discussion to better distinguish between mating potential and actual mating behavior in nature (**lines 770-784**):

"*Our characterization of MAT loci gene content and organization allowed us to infer putative breeding systems (heterothallism vs. homothallism) across species. However, determining how often sexual reproduction and recombination occur in nature, and whether mating involves primarily outcrossing or inbreeding, will require population-level analyses such as linkage disequilibrium decay [140]...*"

We also note in the same section of the Discussion that such analyses remain difficult to implement for many species in our dataset, which are currently known from only a single isolate. Nonetheless, we agree this is a promising direction for future research and appreciate the opportunity to clarify this point in the discussion

Specific comments:

Figure 1: I really like the figure which gives a great overview of the diversity in the groups! Fig 1A: The groups in 1A are are made somewhat arbitrary: F is a paraphyletic group; the line above Kwoniella bestiolae should be below that species; there should be a group added for K. shivajii. Fig 1C: What is meant by "essential"? The fact that many genes are missing in some species suggests these might not be essential. I assume they are only missing from the mating type region, but still present elsewhere in the genome. It might be informative to add this information too.

Response: We thank the reviewer for this helpful feedback. In Fig 1A, the clade groupings were intended to reflect major lineages; however, we agree that the earlier depiction of clade F was misleading due to the line drawn above *K. bestiolae*. Clade F comprises 11 species (14 strains total). We decided to retain the designation "clade E" as a nested subclade within clade F because it aids cross-referencing in the text and supplementary figures and refers to a well-supported cluster of four closely related species (*K. europaea*, *Kwoniella* sp. 1, *K. botswanensis*, and *K. mangrovensis*) that underwent substantial chromosome number reduction.

Regarding gene essentiality (Fig 1C), our intent was to indicate essentiality predictions or experimental validation from *C. neoformans* H99, as noted in the legend and Methods. We agree that the previous wording could be misleading, since absence from the *MAT* locus does not imply absence from the genome.

To clarify both points, we have substantially revised the figure/legend, including removal of the line above *K. bestiolae*, addition of a white asterisk to denote Illumina assemblies (in response to reviewer #2), and correction of a typo (the gene presence frequency plot summarizes panel C, not panel B, as previously stated).

Figure S1: Both trees show a species tree. I wonder if the inconsistency between these trees are due to introgression which is obscured in species trees. Gene trees will most likely show different patters, either due to ancestral polymorphism or geneflow. It might be interesting to test the trees per window or chromosome for example. There seems to be more introgression going on (Line 420, and Fig 5, see the region with reduced variants in the middle clade). It would be interesting to know what is going on genome wide.

Response: We appreciate the reviewer's suggestion. In our dataset, the only notable inconsistency between the concatenation-based tree (S1A Fig) and the ASTRAL species tree (S1B Fig) concerns

the relative placement of *Cryptococcus* clades A, B, and C. This region of the tree exhibits nearly equal support across alternative quartet topologies (with the major topology supported by only ~34% of quartets), which likely reflects limited resolution across a short internal branch rather than true topological conflict. This pattern is more consistent with incomplete lineage sorting than introgression, particularly given the deep divergence among these lineages.

While window-based phylogenetic analyses (per chromosome or sliding window trees) can be informative for detecting localized discordance, they are difficult to implement here due to substantial differences in chromosome composition and synteny across species. Furthermore, many single-gene trees in our dataset likely lack resolution at deeper nodes, limiting their ability to clearly resolve discordant signals. Given these considerations and constraints, and because the topological ambiguity appears localized and is already highlighted in S1 Fig, we consider genome-wide discordance analyses to be beyond the scope of the present study, and hope the reviewer finds this justification reasonable.

We anticipate that improved resolution of these relationships will more likely result from expanded taxon sampling followed by application of methods specifically suited to deep divergences (such as approaches that incorporate gene tree heterogeneity, more explicitly model incomplete lineage sorting, or leverage ultra-conserved elements and rare genomic changes), which we now briefly note in the Discussion in lines **684-686**:

"Further taxon sampling, combined with phylogenomic approaches tailored to deep divergences, will be required to conclusively resolve these relationships and clarify the broader implications of these transitions."

Line 338: "These findings suggest that the lower GC content of the P/R locus is likely driven by a bias towards AT-rich codons, potentially resulting from mutation accumulation associated with reduced or suppressed recombination in this region." This statement is tautological: GC content is lower because AT content is higher. More importantly, the explanation invoking "mutation accumulation" is somewhat misleading. A more likely cause of reduced GC content in regions with suppressed recombination is a decrease in GC-biased gene conversion (gBGC), which is known to be associated with recombination rate (e.g., 10.1186/s13059-014-0549-1). The authors refer to gBGC in the discussion, but only after introducing a long argument about relaxed selection. While relaxed purifying selection may contribute—especially due to reduced effective population size caused by low recombination (e.g., Hill-Robertson interference) and balancing selection—this should not be the default explanation. Before invoking selection-based mechanisms, the authors should consider testing for patterns consistent with neutral processes. For example, examining whether non-coding regions in the P/R locus show similar GC depletion would help determine whether the GC reduction is due to mutational biases rather than relaxed selection. If the effect is genome-wide or evident in non-coding regions as well, a neutral mechanism like reduced gBGC would be a more parsimonious explanation.

Response: We sincerely thank the reviewer for this insightful comment, which prompted us to revisit our analyses of GC content and codon usage in the *P/R* locus with greater precision. In particular, as suggested by the reviewer, we sought to systematically test whether the observed GC depletion reflects reduced GC-biased gene conversion (gBGC), relaxed selection on codon usage, or a combination of both. To address this, we implemented four complementary analyses and briefly describe the main rationale and findings below:

Test 1: Non-coding GC content. We compared GC content of intergenic and intronic regions within the *P/R* locus to length-matched non-coding windows from the same chromosome (excluding the *P/R* region). This directly evaluates the role of gBGC without involving coding sequences. Across all clade D species and several clade B genomes, non-coding GC was significantly lower within the *P/R* locus (permutation test $P < 0.01$), with Z-scores showing strong deviation from chromosomal background. This supports a key role for reduced gBGC in shaping base composition.

Test 2: Synonymous third codon positions. We then quantified GC at synonymous third codon positions (GC3) and compared *P/R* genes to background gene sets of matched length and CDS count. *P/R* genes were significantly enriched for AT-ending codons, consistent with reduced GC content in coding regions.

Test 3: Codon usage bias metrics (ΔENC, Nc, Fop). Subsequently, we calculated several codon bias measures and compared *P/R* genes with background genes of matched GC3. While *P/R* genes showed reduced codon bias, the effect was explained by their altered base composition rather than an independent relaxation of selection.

Test 4: Codon adaptation index (CAI) controlling for GC3 and gene length. Finally, we modeled CAI as a function of GC3 and CDS length and then tested whether *P/R* genes had significantly lower residual CAI than predicted. In most species, no significant residual effect remained, indicating that lower CAI largely reflects neutral processes (e.g., mutation bias or reduced gBGC) rather than relaxed selection.

Taken together, we demonstrate that reduced gBGC in a recombination-suppressed region provides the most parsimonious explanation for the lowered GC content of the *P/R* locus. Apparent reductions in codon usage bias or CAI are consistent with this shift in nucleotide composition, without requiring additional explanations based on relaxed purifying selection.

In revising the manuscript, we also considered the guidance from Reviewer #2, who recommended shortening the main text. For this reason, we now present only a concise summary of the rationale, tests, and key conclusions in the Results (**lines 333-342**), while moving the full methodological details and statistical results to **S1 Text**, and revised **S4 Appendix** and Methods section (**lines 960-1019**).

Line 658: Interesting that the pheromones increased in number. The hypothesis that this might occur for mate finding efficiency has been suggested before. Relevant references: 10.1111/j.1420-9101.2012.02539.x, 10.1098/rspb.2008.1146, 10.1111/jeb.12017. I wonder why this is limited to Cryptococcus. If I understand correctly, this mostly occurs in the mata allele, not the matalpha. Could this in some way be associated with single mating type (unisexual) reproduction? Possibly higher pheromone production in mata is selected to overcome the pheromone independent homothallic mating cycle in the matalpha cells (10.1371/journal.pgen.1006772)?

Response: We thank the reviewer for this thoughtful suggestion and for providing relevant references to guide our interpretation. In the revised Discussion (lines **612-627**), we have now incorporated the studies by Jackson & Hartwell (1990), Rogers & Greig (2009), and Nieuwenhuis et al. (2012) to place the observed pheromone gene duplications in *Cryptococcus* within a broader theoretical framework. These studies suggest that pheromone production may evolve under sexual selection as an honest signal of gamete quality, particularly under conditions of mate competition or limited dispersal, and we have acknowledged this in our interpretation of the data.

However, while *MF* gene expansion is indeed a notable feature of *Cryptococcus P/R* loci, our comparative analyses do not support a consistent bias toward either the *MAT***a** or *MAT*α allele. For example, in the *C. gattii* species complex and *C. neoformans*, *MAT*α typically harbors four *MF* genes, whereas *MAT***a** contains three. In *C. deneoformans*, both alleles carry three copies. Among clade B species, such as *C. amylolentus* and *C. floricola*, *MAT a1* alleles (functionally equivalent to *MAT*α) generally encode four pheromones, while *MAT a2* alleles encode three or four. Similarly, in clade D species, *MAT a1* alleles often carry two *MF* genes, and *MAT a2* alleles four. Given this variation, we do not observe a pattern that would support preferential expansion in *MAT***a**, nor do we find evidence linking these expansions specifically to unisexual reproduction. For these reasons, we have opted not to include further speculation about unisexuality in the main text. Please also refer to a response to reviewer #2 for more details on pheromone gene duplications.

Figure S9 is unclear to me. The colors are not well explained. Are the colors of the chromosomes representing the colors of K. sha? And what about the colors of the centromeres?

Response: We thank the reviewer for raising this concern. In response, we clarified in the revised figure legend that the karyotype of *K. shandongensis* served as the reference in all pairwise comparisons, with each of its chromosomes assigned a distinct color. This color scheme is consistently applied to highlight homologous synteny blocks in the compared *Cryptococcus* species. Centromeres are similarly colored according to their corresponding *K. shandongensis* reference chromosome. In cases where centromeres are inferred to have originated through intercentromeric recombination, two colors are applied to reflect their ancestry from two distinct centromeres associated with different *K. shandongensis* chromosomes.

Figure 10: This figure could be more powerful by simplification. I think the main story is that 1) mat extensions happened multiple times (numbers 2 and 5) and 2) that mat fusions happened multiple times independently (numbers 3, 4 and 6). By giving too many details, the parallelism is lost as the numbers on the tree that refer to these parallel events differ. The evolutionary forces are likely the same for the expansion and the fusions, so I would number them the same.

Response: We appreciate the reviewer's suggestion. However, we chose to number events distinctly to emphasize mechanistic and genomic differences across these transitions. For example, event 2 reflects a small-scale expansion of the *P/R* locus in *Kwoniella*, whereas event 5 marks two independent large-scale *P/R* expansions: one in the common ancestor of clades A, B, and C, and another in clade D. Events labeled as 6 similarly represent independent *HD–P/R* fusion events in *Cryptococcus* pathogens and in *Cryptococcus* sp. 3, which differ in both context and structural outcome. Although these transitions may share similar outcomes (e.g., expansion or linkage/fusion), we retained distinct schematics to highlight the diversity of underlying genomic mechanisms—such as inversion, gene duplication, translocation, and *HD* gene loss. We have updated the figure legend to clarify both the convergent nature of these events and the rationale behind the event numbering.

Reviewer #2's comments:

The manuscript 'Tracing the evolution and genomic dynamics of mating-type loci in Cryptococcus pathogens and closely related species' by Coelho and colleagues reports on a comprehensive study of the mating type evolution in two genera of basidiomycete fungi. By taking advantage of existing and novel genomic resources, the authors perform comparative genomics to uncover the genome dynamics of mating type loci, resulting in transitions in reproductive strategies, associated with genetic and chromosomal reorganizations at the mating type loci.

This submission extremely comprehensive and collects a large body of exciting and novel data. It is generally well written, and the analyses are generally performed to high standards, using state-of-the-art approaches. The results are presented clearly, figures are of high quality; the overall robustly support the manuscript's conclusion. To accommodate the large body of analyses and results, the manuscript is extremely long with and extensive number of (complex) figures. At the same time, the manuscript's text is also at times challenging to follow for a non-expert in fungal mating and genomics and to pick out what are the key findings. To maybe appeal better to the broad readership of PLoS Biology and to generally improve clarity, it would be beneficial to shorten and focus the manuscript on fewer key observations and either remove others (e.g., coding adaptation, or the in-depth discussion of additional genes associated with mating type loci) from the manuscript or move to S-Material/Text; this is up to the discretion of the authors and the editor. We have also provided additional detailed comments and suggestions aimed at improving the overall clarity and at addressing few remaining open points of concern:

Response: We thank the reviewer for his thoughtful assessment and positive comments on the scope and quality of our analyses. We appreciate the concern, shared by three reviewers, that the manuscript is long. In revising the manuscript, we have streamlined several sections and moved some of the more technical or detailed aspects to S1 and S2 Texts. Below, we address each of your additional comments and outline the revisions made to our manuscript accordingly. Please note that line numbers refer to the revised clean manuscript without tracked changes.

L98: Dikaryotic hyphal growth is a process already regulated by the canonical HD genes (homeodomain transcription factors) in the MAT locus. HD genes are not 'additional genes', as these are core of the mating type system in all basidiomycetes with a tetrapolar or bipolar mating system. Therefore, attributing dikaryotic growth to the effect of additional genes recruited into fused MAT loci is misleading or imprecise, and this sentence needs to be rephrased.

Response: We thank the reviewer for pointing this out. To clarify, our original statement was not referring to the canonical *HD* genes (which are indeed core components of the *MAT* locus and key regulators of dikaryotic development in basidiomycetes) but rather to additional genes that have become entrapped in fused *MAT* locus over evolutionary time. For example, *STE20*α, located within the fused *MAT*α locus of *C. neoformans*, is dispensable for initial mating partner recognition but required for proper polarity maintenance during dikaryotic hyphal growth (Nichols et al., 2004; PMID: 15282344). We have revised the text accordingly to more accurately reflect this distinction and to avoid any potential confusion. The sentence now reads (**lines 95-98**):

An important aspect of fused MAT loci is the inclusion of genes beyond the core HD and P/R determinants, some encoding regulators of post-mating development that influence morphogenesis (e.g., STE20, required for polarity maintenance in dikaryotic hyphae [36]) and dispersal (e.g., SPO14, likely involved in spore production) [37].

L157: It would be helpful to clarify whether the sequenced genomes are haploid or dikaryotic. If they are dikaryotic, were the genome assemblies phased? This information is important for interpreting the assembly and analysis results. For example, given that CBS10435, a parental strain used in crosses (Fig. 9C), was found to be diploid (L608), it would be helpful to clarify whether ploidy was confirmed for the other sequenced isolates to ensure consistency in the comparative analyses.

Response: We thank the reviewer for this point. We have now clarified the ploidy status of the sequenced genomes in the revised text (**lines 164-165**), which now reads:

"Analyzed strains were inferred to be haploid based on genome sizes consistent with those previously documented for haploid strains [49]".

Additional support for this includes the presence of a single *MAT* allele per strain, as detailed later in the manuscript. The only exception is strain CBS10435, which we later show to be diploid based on FACS analysis. No other strain showed evidence of diploidy.

L162: How were the mating types determined to guide the strain selection? Was this done prior to the analyses, based on previous work, or indicated based on observations from this submission. This should be clarified.

Response: Good point. We have now clarified the basis for mating type assignment in the revised text (**lines 156-160**), which now reads:

For Kwoniella, three strains belong to the recently described species K. ovata, K. endophytica, and K. fici [48, 70, 71], while the remaining four strains are different mating types of K. mangrovensis, K. europaea, K. botswanensis, and K. heveanensis, as previously determined through PCR and mating tests [52, 66, 67].

L202: In Figure S2A, the individual cultures of C. decagattii strains are shown plated alone, but the yeast morphology is not clearly depicted. For clarity and consistency, it would be helpful to include representative images or insets of the yeast cells for the single-strain cultures, to fully illustrate the yeast-to-hypha transition described in the text; this also applies for Figure S2B. In Figure S2C, for C. depauperatus, it would be helpful to clarify what is shown in the zoomed-in inset for the YPD medium — is this hyphal growth or basidia? Additionally, in the V8 pH 5 condition, the structure appears to be a spore chain, but the text also mentions basidia formation. Basidia are not clearly visible in the current image, it would strengthen the figure to include an additional inset specifically highlighting the basidia.

Response: Thank you for these helpful suggestions. We have revised the S2 Fig and legend as follows:

1. Panels A and B (*C. decagattii* and *C. tetragattii*): we added higher-magnification images of yeast cells from the solo cultures of each strain. Instead of presenting these as small insets, we now provide larger right-panel images to highlight yeast morphology and illustrate the contrast with hyphal growth observed in the *C. decagattii* cross. For the *C. tetragattii* α × **a** cross, no hyphae or sexual structures were formed, and the culture remained only a mixture

of yeast cells. Since this would be redundant with the solo culture panels, we did not include additional yeast cell images for the cross.

2. Panel C (*C. depauperatus*): we clarified in the legend that the right panel for the YPD condition shows hyphal filaments rather than basidia. We also revised the V8 pH 5 zoomed panel by explicitly indicating the basidia (arrowheads) and spore chains (brackets) in the higher-magnification photo.

3. Figure legend: we fully revised the legend to explicitly state that left images show the colony edge and right images show higher-magnification views of yeast, hyphae, or sexual structures from the same culture. Scale bar information was also harmonized across panels.

L227: Few strains were sequenced with Illumina only, resulting in fragmented assemblies. How does this impact the manuscript's results on sizes of mating type loci, organization and distances between genes? It might be useful to indicate genome assembly quality (i.e., which are chromosome-level or not) in Figure 1.

Response: We thank the reviewer for raising this point. In response, we have updated Fig. 1A to indicate with a white asterisk (explained in the revised figure legend) that *K. europaea* PYCC6162, *K. botswanensis* CBS12717, and *K. heveanensis* BCC8398 were sequenced exclusively with Illumina. While this information was already stated in the text, we agree that including it directly in the figure facilitates cross-referencing.

Despite the more fragmented nature of these three assemblies, the *P/R* and *HD* loci were fully recovered in all cases, as shown in Fig. 2 (for *K. heveanensis*) and S4 Fig. (for *K. europaea* and *K. botswanensis*). This allowed confident inference of *MAT* locus gene content, size, and organization. We think this was feasible with Illumina data alone because the *P/R* locus in these species has a relatively simple architecture, containing only a single pheromone gene.

In addition, for each of these species, the opposite mating type has been sequenced to chromosome-level, telomere-to-telomere completeness, either in a previous study (Coelho et al., 2024) or in the current study (e.g., *K. mangrovensis* CBS8886). These assemblies confirm a tetrapolar organization and provide a reliable basis for comparative analyses. Given this, and the general conservation of tetrapolar configurations across most *Kwoniella* species, we consider it highly likely that the strains represented only by Illumina-based assemblies retain the same overall *MAT* structure.

L259 (and other locations, e.g., L320): How were the different mating-associated loci defined? For example, by considering all genes flanked by known mating type genes, or differently? This is obviously relevant for i) determining the size and ii) determine the genes localized within a specific locus. Moreover, there might be genes relevant for mating just outside of the loci, that are currently not considered. The manuscript could be more explicit when it comes to the approach and implication of the mating locus definition. Moreover, in L439 it is mentioned that the two most distal pheromone genes were considered to determine the length. Shouldn't be the minimum distance covering HD genes, STE3, and MF genes be considered, and thus the length should cover both loci.

Response: We appreciate the reviewer's comment. We have now clarified in the Results section (lines **230-232**) that: *"The HD locus was defined based on the presence of the HD1 and HD2 genes,*

while the boundaries of the more variable P/R locus were delineated by identifying regions of disrupted synteny between mating types (see Material and Methods).

In the Materials and Methods (**lines 918-923**), we further explain that for species with only one mating type available, the *P/R* locus was inferred from the region between conserved flanking genes, using comparisons with opposite mating types from closely related species.

We acknowledge that precise delineation of the *P/R* locus would ideally require genome assemblies for both mating types of each species. However, this is not currently feasible for many *Kwoniella* species, which are known from only a single isolate. As such, we think our approach for defining the *P/R* locus boundaries based on synteny provides a consistent and standardized framework.

We also indicate instances of uncertainty in Fig 1C, where a few genes of unclear locus affiliation are shown in dark grey. These represent genes that may belong to the *P/R* locus in certain tetrapolar *Kwoniella* species, or to the fused *MAT* locus in bipolar species, but for which confident assignment is not possible due to limited synteny or lack of an opposite mating type. Such genes were classified as “unclear” and, as indicated, were not included in *MAT* locus size calculations or gene count summaries.

Lastly, regarding the reviewer’s example, we agree that *Cryptococcus* sp. 3 (strain CMW60451) represents an exceptional case. As only one strain is available, we report a conservative minimum *MAT* locus length based on the span between the two most distal pheromone genes, which are expected to reside within the fused *MAT* region. The additional genes mentioned by the reviewer (*STE3* and *HD/SXI2*) are in fact embedded within this interval between the distal pheromone genes. A broader estimate, extending to additional genes typically included in the *MAT* region of pathogenic *Cryptococcus* species (e.g., *RPL22*, *STE12*), is also provided and clearly distinguished in both the text and Fig 6C.

L275: 'a species' -> "Within Kwoniella species…"?

Response: Corrected as suggested.

L284: How were the rearrangement breakpoints determined? By manual evaluation of gene localization, nucleotide alignments, or …? Could you please clarify this?

Response: We thank the reviewer for this question and the opportunity to clarify. The approximate rearrangement breakpoints shown in S5 Fig were inferred through synteny comparisons using BLASTN (threshold: >0.1 kb alignment length), combined with manual evaluation of gene order and orientation between the *P/R a1* and *a2* alleles. Specifically, we used conserved syntenic blocks and the presence of gene remnants—such as partial fragments of *NDC80* and *BSP3*—detected at the inferred inversion edges in *P/R a2* alleles of clade E/F and clade G species. These features allowed us to reconstruct the likely series of rearrangements by inverting gene blocks in the *a2* alleles to recapitulate the configuration found in the *a1* alleles, which we assume to reflect the ancestral state. This assumption is supported by the high conservation of gene order and orientation across all analyzed *a1* alleles within and between *Kwoniella* species, in contrast to the *a2* alleles, which exhibit clade-specific inversion patterns. While we cannot rule out the possibility that *a1* alleles may have also undergone rearrangements during evolution, the available comparative evidence suggests that the inversions predominantly affected the *a2* alleles.

We have clarified this inference approach in the revised Methods section (**lines 924-930**), where we now state:

*"To infer the origin of structural rearrangements observed in the P/R locus in Kwoniella (**S5A Fig**), we manually inspected the orientation and synteny of P/R-associated genes across mating types and species. Rearrangement breakpoints were approximated by identifying changes in gene order and orientation between alleles, supported by the presence of gene fragments (e.g., partial NDC80, STE12, BSP3) and conserved synteny in the opposite mating type. These rearrangements were reconstructed by inverting or repositioning affected gene blocks in the more derived a2 allele to match the putative ancestral configuration in the more conserved a1 allele."*

L339: it is possible that the deviation of AT-rich codons is a signal of introgression rather than an adaptation specific at the MAT locus. Are there additional genomic regions that would show similar deviations (the manuscript reports for enrichment analysis compared to the genome-wide numbers)? Does the phylogeny of genes in MAT loci follow the general species phylogeny? Moreover, in the discussion (L690 and following), the authors later acknowledge that codon usage and expression analyses are needed to better understand P/R locus evolution, in this paragraph they present speculative interpretations about codon usage effects on mating without direct supporting data for the P/R locus itself. I suggest clarifying within this paragraph that these interpretations remain hypothetical and would benefit from future experimental validation.

Response: We thank the reviewer for these thoughtful comments. Considering your feedback, and a similar point raised by Reviewer #1, we expanded our analyses of GC content and codon usage at the *P/R* locus to better distinguish neutral from selective explanations (see detailed response to Reviewer 1 above). These analyses, now briefly described in the result section (**lines 334-341**) and further extended in **S1 Text** and the revised **S4 Appendix**, support reduced GC-biased gene conversion (gBGC) in the recombination-suppressed *P/R* locus as the most parsimonious explanation for its AT-rich composition. Consequently, we have removed relaxed selection on codon usage as a required explanation and substantially revised the Discussion (**lines 646-657**) to focus on reduced gBGC as the primary mechanism.

With respect to the reviewer's suggestion of introgression, we also consider this unlikely for several reasons. First, both *a1* and *a2 P/R* alleles are AT-rich in clade C and D species. Explaining this by introgression would require two independent and coordinated events, which is less parsimonious than a shared locus-specific process. Second, the GC depletion extends into introns and intergenic regions. While an introgressed block could in principle include both coding and non-coding sequence, the consistent depletion across multiple species would require long-term persistence of the same introgressed haplotype, which is difficult to reconcile with the data. Third, our prior phylogenetic analyses (Passer et al. 2022; PMID: 35713948) showed that most *P/R* genes follow the species phylogeny, with only a subset displaying deep trans-specific polymorphism. We did not observe a coherent block of incongruent trees that would be expected if the entire locus had introgressed from another lineage.

L483: could the manuscript at this stage provide an intuition how the centromeric positions had been determined? Experimentally using ChIP or computationally using signature transposons/repeats?

Response: Thank you for this suggestion. However, while streamlining the manuscript, the detailed description of centromere inference and loss in *Cryptococcus* sp. 3 has been moved to **S2 Text**, where we indicate that:

"Centromere positions were inferred computationally based on the presence of characteristic long terminal repeat (LTR) elements typically enriched at Cryptococcus centromeres, in combination with synteny comparisons and identification of the longest ORF-free intergenic regions [1-4]."

In the Methods section (**lines 914-916**), *we further clarify that "centromere positions were determined in silico as previously described [49], combining detection of centromere-associated LTR elements and synteny analysis. Centromere lengths were defined as the intergenic regions between flanking centromeric genes (S1 Appendix)"*.

L623: while typically not done, it might be useful given the amount of data and length of manuscript to cross-reference between the discussion and figures to enhance readability.

Response: We appreciate this suggestion. In the revised Discussion, we reference Fig 10 as a roadmap for the major evolutionary events, which we think provides readers with a visual guide to follow the broader narrative. We considered adding additional cross-references to specific figures, but given the already extensive length of the Discussion, we opted to instead streamline the text for improved flow and readability. We hope that the revised structure, together with Fig 10 as an integrative reference point, now makes the section easier to follow without needing multiple figure callouts. However, we are happy to revisit this if the editor or reviewers feel they would substantially enhance readability.

L634/658 (and other): Much of the discussion on mating type systems and their dynamics is focused on basidiomycete yeasts, but little attention is given to rust fungi where recently several manuscripts report about the composition and evolution of mating type loci, with striking analogies to results reported in this manuscript. For example, multiple MF gene copies have been described in these rusts, which should be discussed here. Moreover, the authors suggest that pheromone duplications evolved to strengthen mating partner recognition. However, it would be useful to clarify whether functional validation exists for all the duplicated pheromone genes, specifically, whether they are all actively expressed and contribute to mating compatibility. If not experimentally confirmed, I recommend rephrasing this statement more cautiously. The manuscript also reports multiple STE3 genes in the P/R loci. It would be helpful to clarify whether any of these duplicated genes are known or suspected to be functionally active in mating compatibility, and whether one or several copies are likely to be primarily responsible for partner recognition.

References of MAT loci in rust fungi:
1. Gomez-Zapata, P. A., Tellgren-Roth, C., Samils, B., Stenlid, J., Kaitera, J., Brandstrom Durling, M., ... & Olson, A. (2025). Mating Type Gene Divergence is Associated with Life Cycle Differentiation in Scots Pine Blister Rust. bioRxiv, 2025-05.
2. da Rocha, V. D., Ferreira, E. G. C., Castanho, F. M., Kuwahara, M. K., Godoy, C. V., Meyer, M. C., ... & Marcelino-Guimarães, F. C. (2025). Analysis of the genetic diversity of the soybean rust

pathogen Phakopsora pachyrhizi reveals two major evolutionary lineages. Fungal Genetics and Biology, 103990.
3. Sperschneider, J., Chen, J., Anderson, C., Morin, E., Zhang, X., Lewis, D., ... & Dodds, P. (2025). A chromosome-scale genome assembly of the flax rust fungus reveals the two unusually large effector proteins, AvrM3 and AvrN. bioRxiv, 2025-04.

Response: We appreciate the reviewer's thoughtful suggestions. We recognize that one challenge in revising the manuscript has been reconciling feedback to keep the text concise with requests to expand specific areas of the Discussion. In light of this, we chose to incorporate the reviewer's points in a targeted way that acknowledges relevant parallels without substantially lengthening the manuscript.

Accordingly, we have expanded the Discussion to briefly reference recent work on *MAT* loci in rust fungi (**lines 587-586**), particularly highlighting the reports of multiple *MF* pheromone genes and *STE3* receptor duplications in species described by Gómez-Zapata et al. (2025) and Sperschneider et al. (2025). These studies were released after our initial submission and therefore had not been included previously. While they reveal intriguing parallels to patterns we describe in *Cryptococcus*, we note that the genomic structure and boundaries of the *P/R* locus in rust fungi remain incompletely resolved, partly due to genome complexity and limited availability of phased assemblies.

Regarding the functional role of pheromone duplications, we now cite the experimental study by Shen et al. (2002) (PMID: 12455985), which showed that all three *MF*α copies in *C. deneoformans* are expressed and contribute additively to mating and filamentation. This supports a dosage-based model in at least one species. However, as functional data from other species are lacking, we have revised our statements to present the adaptive role of *MF* duplications as a working hypothesis rather than a general conclusion (**lines 612-617**).

With respect to *STE3* receptor duplications, we note that our results show that each mating type in *Cryptococcus* and *Kwoniella* encodes a single full-length *STE3* gene. The only apparent exceptions are *K. ovata* and *K. endophytica*, which contain truncated remnants of a second *STE3*α-like gene adjacent to a full-length *STE3***a**. As discussed in the manuscript, we interpret these as likely products of intra-locus recombination rather than functional duplications. Their functionality remains uncertain, and while expression analyses would be informative, the lack of available opposite mating-type strains for these species currently precludes experimental validation.

L803: The authors might consider discussing the potential evolutionary link between the transition to bipolar mating systems and the emergence of pathogenicity in Cryptococcus species, as all known pathogenic species are bipolar.

Response: We thank the reviewer for this thoughtful suggestion. We have now expanded the Discussion (**lines 750-767**) to address the possible relationship between bipolarity and pathogenicity. While all currently recognized pathogenic *Cryptococcus* species are bipolar, our results also reveal fused *MAT* loci in non-pathogenic species such as *Cryptococcus* sp. 3 (isolated from bark beetles) and *Kwoniella fici* (isolated from fig). In the Discussion, we note that this indicates bipolar organization of *MAT* can evolve independently of pathogenicity, plausibly in response to restricted partner availability in specific ecological settings. At the same time, once *MAT* linkage becomes established, the resulting bipolar state may promote its long-term maintenance and promote conditions that could later support the evolution of pathogenic lifestyles.

L922: could you provide some details how each round of error correction impacts the errors, as reported by pilon. It is possible that an optimal correction is already achieved after fewer iterations and additional rounds of error correction will actually reduce the quality of the final assembly.

Response: Thank you for raising this concern. As noted, we used Pilon to iteratively polish the assemblies but halted the process when no further corrections were reported by the software. Specifically, we implemented a conditional loop that checked for the presence of a non-empty <*.changes*> file after each round. If no changes were detected in a given iteration, the process was automatically stopped, thus minimizing the risk of overcorrection. This approach ensures that the optimal number of iterations was used for each assembly individually, as the number of rounds required varied depending on the starting quality of the input assembly. While we did not systematically track and report the exact number of corrections per round for each genome (given the scale of our dataset), the use of Pilon's built-in convergence logic provided a practical and robust safeguard against diminishing returns in polishing accuracy. We have now clarified this in the revised Methods section (**lines 831-833**).

L941: How is the manual annotation performed, i.e., how were the gene models amended? Is this annotation publicly available?

Response: Manual annotation of mating-type genes was performed by comparing gene predictions and alignments to known *Cryptococcus MAT* gene orthologs. Gene models were amended by adjusting exon–intron boundaries and by adding small ORFs missed by automated pipelines, including the short *MF* pheromone precursor genes. These curated annotations were incorporated into the final GFF files and will be made publicly available as part of the genome submissions currently being processed at NCBI. This is now explicitly stated in the Methods section (**lines 852-855**).

L1743: What are the gCF/sCF values for the other branches? Are these branches well supported?

Response: We appreciate the reviewer's attention to this detail. gCF and sCF values are shown for all branches in S1 Fig, along with SH-aLRT and UFBoot support. While values vary across the tree, most branches exhibit substantial support. As expected in a dataset spanning both shallow and deep divergences, some internal nodes (particularly those involving recently diverged taxa, such as within the *C. gattii* species complex) show lower concordance, possibly reflecting incomplete lineage sorting. However, the tree topology from the concatenation-based analysis (S1A Fig) is largely congruent with the ASTRAL coalescent species tree (S1B Fig). The only notable discrepancy is the relative placement of clades A, B, and C. This region also exhibits lower quartet support in the ASTRAL analysis, with the q1 value (~0.34) only marginally above the 0.33 threshold expected under a hard polytomy. All other internal branches have q1 values above this threshold, suggesting a well-resolved topology overall. While a full resolution of these relationships is beyond the scope of the current study, improved phylogenetic clarity will likely require expanded taxon sampling as now stated in **lines 684-868**. We chose to display the full concordance and support values in S1 Fig, rather than in the main figure (Fig 1A), to maintain visual simplicity, while indicating the ambiguous placement of clades A, B, and C with dashed branches.

Figures: it is confusing that the tree in Figure 1a has similar labels as the panels (A-I).

Response: Thank you for this observation. We recognize that the reuse of letters may be visually confusing; however, the labels A–I in Fig 1A refer to major clades defined in our phylogeny and are consistently used throughout the manuscript to support comparisons and facilitate cross-referencing across figures and sections. These clade labels are embedded directly within the tree, formatted in standard font and size. In contrast, figure panel labels (A–E) are presented in bold and larger font, following standard journal formatting, to distinguish them clearly as panel identifiers. If acceptable to the reviewer, we would prefer to retain the current clade designations for consistency across the manuscript.

Reviewer #3's comments:

The manuscript by Coelho et al. uses a wide variety of genomic, evolutionary, and mating experiments to reconstruct the evolution of the mating-type loci in the Cryptococcus-Kwoniella clade and, to a certain extent, the evolutionary steps that gave rise to the observed genomic architectures of the mating loci observed in the different species. It is a tour de force that will be widely read and cited by any biologist interested in the evolution of fungal mating systems. The manuscript is long and on the descriptive end of things; that said, it is very clearly written and I had no trouble following the authors' experiments and stream of presentation.

Response: We thank Reviewer #3 for careful reading of our manuscript and for their supportive and constructive comments. We are grateful for the positive evaluation, particularly noting the clarity of the manuscript and the significance of our findings for understanding the evolution of fungal mating systems. We address your specific comments below and outline the revisions made. Line numbers refer to the revised clean manuscript without tracked changes.

To me, the highlight of the story is the discovery of an independent fusion event of the P/R and HD loci is Cryptococcus species 3 and the independent tetrapolar to bipolar and pseudobipolar transitions in Kwoniella. I share the authors' feeling that the more we sample the diversity of these fungi, the greater the insights into the evolution of these highly dynamic loci will be. By "sampling" the authors seem to focus only sampling additional species, but my sense is that population sampling (especially in instances where populations are structured) will also be of help and may help identify - if they get lucky - potential transitions in the making. Not sure how amenable species in the two genera are for population genomics (especially outside of the pathogens), but it would be worth commenting on this somewhere in the discussion.

Response: We thank the reviewer for this fair assessment and fully agree that broader population-level sampling could provide valuable insights into transitions in mating system organization. At present, however, such efforts are constrained by the fact that, outside of the pathogenic clade (where multiple isolates per species are often available), most non-pathogenic *Cryptococcus* and *Kwoniella* species are known from only one or a few isolates, limiting the feasibility of comprehensive population genetic studies. While we had already highlighted the importance of diversity sampling in the Abstract and Discussion, we have now more explicitly addressed the need for population-level sampling across multiple species in the revised Discussion (lines **770-784**).

I also found it very intriguing that the two switches from tetrapolarity to bipolarity in Cryptococcus took place at or very near the branch that is hard to resolve (the one concerning relationships of clades A, B, and C). Perhaps the mutational events that gave rising to bipolarity in clade A (and perhaps C) also contributed to the reproductive isolation of the three lineages? Are all three topologies roughly equally likely? (i.e., is support for A+B roughly equal to support for A+C as well as to support for B+C?) I know that it is hard to test this hypothesis, but one might expect that such transitions are often accompanied by reproductive isolation of strains with different genomic organizations of the mating locus? Might be worth speculating on this as well.

Response: We appreciate the reviewer's point. The phylogenetic branch connecting clades A, B, and C remains difficult to resolve. In our ASTRAL analysis, the three possible topologies received nearly equal quartet support (q1 = 0.34, q2 = 0.34, q3 = 0.32), indicating that alternative relationships among these clades are similarly plausible. In our previous study with fewer species (Coelho, 2024 PMID: 38843310), we recovered a topology consistent with the main ASTRAL placement, but overall support was also weak. We therefore consider this branch unresolved at

present, and additional sampling of lineages bridging pathogenic and non-pathogenic taxa will likely be required to clarify these relationships.

In the current study, our data suggest that the transition to bipolarity in pathogenic *Cryptococcus* likely occurred independently from that in *Cryptococcus* sp. 3. However, it remains uncertain whether the initial translocation event that brought the *P/R* and *HD* loci onto the same chromosome occurred independently in these lineages or in their last common ancestor. We have highlighted this uncertainty in the Discussion (**lines 674-686**). While it is possible that such structural changes at *MAT* may have contributed to reproductive isolation between clades, the absence of robust phylogenetic support currently prevents us from drawing strong conclusions, and we therefore we would prefer not to speculate further on this point.

Minor comments:

- Line 183-184: "forming an early-branching clade": the term "early-branching" is inaccurate - better to phrase this in terms of sister relationships. Maybe "forming a clade that is sister to the rest of the species in the genus Cryptococcus"?

Response: We thank the reviewer for the suggestion. This has been revised to read:

"Cryptococcus sp. 4 [72] and Cryptococcus sp. 5 clustered with Cryptococcus sp. 6 (OR918) [49, 73], together forming a sister clade to all other known Cryptococcus."

- Line 228: "loci" -> "locus"

Response: We agree with the reviewer that the original phrasing ("each *MAT* loci") was awkward. In this case, we were referring to both the *P/R* and *HD* loci across species, so the plural form seemed appropriate. To improve clarity, we have revised the sentence (**lines 226-227**) to read

"These findings prompted detailed characterization of MAT loci across species and reconstruction of the chromosomal changes underlying bipolar configurations."

- Lines 677-692: relaxation of selection could be tested with dn/ds types of models implemented in software such as PAML and HyPhy.

Response: Thank you for this suggestion. The section cited has been substantially revised following our expanded analyses of GC content and codon usage at the *P/R* locus (see responses to Reviewers #1 and #2; S1 Text and revised S4 Appendix). These analyses indicate that reduced gBGC, likely resulting from recombination suppression, is the most parsimonious explanation for the AT enrichment of the *P/R* locus of clades C and D, and we no longer invoke relaxed codon usage selection as a necessary explanation.

Additionally, if we understand the reviewer correctly, the suggestion to use dN/dS models refers to testing relaxation of selection at the protein level. Our original discussion in this section, however, focused on selection on codon usage, not amino acid substitutions. While dN/dS methods in PAML or HyPhy are powerful for detecting relaxation of selection on protein-coding changes, our understanding is that they are not designed to test hypotheses about codon usage bias at

synonymous sites. For this reason, and in light of our reanalysis, we revised the Discussion (**lines 646-657**) to emphasize reduced gBGC as the primary driver of GC depletion at the *P/R* locus.

- Line 819: "most definitive" -> "definitive"

Response: Removed after revising the corresponding section.

- Line 820 onward: I agree that sampling of new species will be useful. In addition, the authors might want to add a note about the importance of population sampling (especially in species with evidence of structured populations). These genomic structural rearrangements must have come from somewhere and sampling at the population level might enable catching such a rearrangement "in the act" so to speak.

Response: We thank the reviewer for reiterating this important point. As noted above, we fully agree that population-level sampling would be particularly valuable, as it could reveal the origin and spread of genomic rearrangements and perhaps even capture such transitions in progress. We have included this discussion point in **lines 770-779**.

- Figure 10: why invoke two independent transitions to step #5 and not a single one in the branch subtending the two with transitions to step # 5? It's more parsimonious that way

Response: We thank the reviewer for this perceptive comment. Step 5 refers specifically to *P/R* loci expansions. While a single expansion event at the node subtending clades B and D would appear more parsimonious, the underlying gene content indicates otherwise. It is possible that an initial expansion of the *P/R* locus—incorporating a subset of additional genes beyond the ancestral *STE3* receptor and pheromone (e.g., *STE20, STE12, BSP3, MYO2, RPL39*)—occurred prior to the divergence of clades B and D. However, the subsequent large-scale expansions in each clade are characterized by distinct gene acquisitions, duplications, and rearrangements that are not shared across lineages. Clade B species retain some *P/R* genes that in clade D are absent or located in the flanking regions, whereas clade D species harbor up to ~15 unique genes within the *P/R* locus that are not present in clade B. These differences are most consistent with independent expansions in clades B and D, even if both may have built upon an earlier, smaller-scale locus expansion. We have clarified this interpretation in the Results (**lines 302-326**), Discussion (**lines 602-604**) and figure legend.

Reviewer #4's comments:
[identifies himself as Aaron A. Vogan]

The manuscript "Tracing the evolution and genomic dynamics of mating-type loci in Cryptococcus pathogens and closely related species" by Coelho et al. is a comprehensive investigation into the genome evolution of species of the genera Kwoniella and Cryptococcus, with a focus on the MAT locus. This is an excellent manuscript, well written, clear and encompassing.

Response: We thank the reviewer for taking the time and effort necessary to review the manuscript and for the encouraging comments. Below, we address your specific comments and outline the revisions made to our manuscript. Please note that line numbers refer to the revised manuscript without tracked changes.

I have only 1 comment that I feel should be addressed before publishing. This relates to the analysis of introgression of the MAT locus for the VNI strains. The trees are showing the topology of 3 clades to draw conclusion about sister relationships, but this is not possible with only 3 tips. Proper outgroups are required, or more information about how the root was placed. Additionally, it would be more informative to have trees of genes flanking the MAT locus, rather than the whole of chromosome 5 to compare to see how quickly the signal of introgression disappears outside of the MAT locus.

Response: We appreciate this thoughtful comment. For the VNI/VNBI/VNBII trees, we used midpoint rooting, as stated in the figure legend. We considered using an outgroup; however, the closest available (*C. deneoformans*, ~12% divergent from *C. neoformans*) is sufficiently distant that outgroup rooting would greatly reduce the shared SNP set and potentially erode resolution at the VNI–VNBI–VNBII split. For this reason, we consider midpoint rooting is the most appropriate and transparent choice in this case.

Regarding flanking regions, we agree that assessing how the introgression signal decays away from *MAT* is informative. As shown in Figure 5B, the signal is largely confined to MAT, fading rapidly on the right and limited to only a few genes on the left. We also now cite prior work mapping the upstream boundaries of this introgression (Desjardins et al., 2017, PMID: 28611159), which is consistent with our observations. Our aim here was not to re-establish the introgression event itself, but rather to highlight novel synteny differences within the *MAT***a** region across VNI, VNBI, and VNBII, which we interpret as more compatible with post-introgression rearrangements within *MAT*. To avoid redundancy and maintain focus, we would prefer not to include additional gene trees, but we have clarified in the text and legend that the introgression signal is local to *MAT* and aligns with previous boundary estimates and is consistent with recombination hotspots flanking the locus (**lines 384-386**).

Beyond this I have noticed a very minor issues that can be addressed
LINE 120: Can one forage for mating partners?

Response: We thank the reviewer for pointing this out. We agree that the use of "foraging for mating partners" was misleading in this context, as the evidence for hyphal growth facilitating partner searching primarily pertains to unisexual reproduction, rather than **a**–α mating. To avoid overinterpretation, we have revised the sentence to remove "and mating partners".

The updated sentence now reads (**lines 116-118**):

"This morphological switch aids nutrient foraging [56, 57] and enhances survival against environmental stressors and microbial predators, thought to be major selective forces in Cryptococcus evolution [53, 58-62]."

LINE 156: Say how big the previous data set was

Response: We appreciate the suggestion. In our previous study (Coelho & David-Palma et al. 2024), we analyzed 29 *Cryptococcus* and *Kwoniella* genome assemblies (including 22 newly sequenced). In the present study, we incorporated high-quality assemblies for 19 additional strains, several of which represent the opposite mating type of species previously analyzed. We now clarify this in the revised text (**lines 151-153**) as follows:

"To enable detailed analyses of MAT locus structure and evolution, we expanded our previous genomic dataset (29 genome assemblies; [49]) by incorporating genome sequences for 12 additional Cryptococcus and 7 Kwoniella strains, including opposite mating types where available".

LINE 164: One doesn't assemble by long- and short-read sequencing. You generate data form sequencing and then assemble. It should also be clear if this is co-assembly with Illumina or assembly followed by polishing.

Response: Good catch! We acknowledge the ambiguity and have amended the phrasing accordingly (**lines 160-162**). The updated text now reads:

"All but three genomes were assembled from long-read sequencing data (PacBio or Oxford Nanopore) and polished with short-read (Illumina) data, yielding chromosome-level genome assemblies".

Paragraph at 294: Does this suggests a transition from heterothallic to homothallic and back to heterothallic? The transition from homothallic to heterothallic had been considered unlikely and/or unfeasible traditionally, very interesting observation.

Response: We thank the reviewer for highlighting this interesting point. While our data suggest that the extant *P/R* locus in *K. ovata* and *K. endophytica* may have originated through intra-locus recombination between *a1* and *a2* alleles, potentially enabling self-filamentous growth, we do not interpret this as evidence of a full transition to homothallism. Rather, these recombinant configurations may have conferred transient self-compatibility at the *P/R* locus within a single nucleus, but were likely followed by pseudogenization of *MF*α and *STE3*α, resulting in a reversion to strict heterothallism. For a complete transition to homothallism, both compatible *HD1* and *HD2* alleles would also typically need to be present in the same genome, either through physical linkage or mutations that bypass the usual requirement for non-self-recognition between *HD* partners. Notably, the only known primary homothallic species in *Cryptococcus* is *C. depauperatus*, which appears to have evolved self-compatibility through a distinct route. In this species, we previously identified a haploid genome containing *STE3***a** and *MF*α alleles, but lacking homologs of the canonical *HD* genes (*SXI1* and *SXI2*), suggesting that compatible pheromone and receptor gene pairs may alone be sufficient to drive homothallic behavior. This was reported in Passer et al. (2022, *eLife* 11:e79114). We now clarify these points in the revised Discussion (**lines 596-600**).

FIGURE 6B The C. neo chr_5 and C. sp3 *Chr_11 comparisons show identical chromosome maps on the left and right, but very different synteny. Why?

Response: We understand the reviewer's concern. To clarify, in panel B, *C. neoformans* chr. 5 and *Cryptococcus* sp. 3 chr. 11 are indeed shown twice. This is because, in bipolar species, the *P/R* and *HD* loci are located on the same chromosome. To allow direct comparison with tetrapolar species, where these loci reside on different chromosomes, we chose to represent the chromosome twice: on the left aligned to the *P/R*-containing chromosomes of other species, and on the right aligned to the *HD*-containing chromosomes. Although the chromosome maps are identical, the anchor point for each synteny comparison differs, resulting in distinct flanking block patterns.

To make this clear, we have revised the figure legend to state:

"(B) Linear chromosome plots depicting gene synteny conservation across species with zoomed-in views depicted in (C). In panel B, the MAT chromosome of bipolar species (C. neoformans and C. sp. 3, CMW60451) is shown twice: the left column depicts synteny relative to the P/R-containing chromosomes of tetrapolar species, and the right column depicts synteny relative to the HD-containing chromosomes".

LINE 466: How do gene phylogenies reflect rearrangements?

Response: We appreciate the reviewer's query. In this section, the gene phylogenies do not directly reveal chromosomal rearrangements. Rather, they clarify whether the physical linkage of the *P/R* and *HD* loci in *Cryptococcus* sp. 3 likely arose through an independent fusion event or was inherited from a shared ancestor with pathogenic *Cryptococcus* species. By examining the genealogies of four genes (*BSP3*, *PRT1*, *SPO14*, and *CAP1*) located within the fused *MAT* region and known to exhibit mating-type–specific clustering in pathogenic species, we found that *Cryptococcus* sp. 3 sequences did not group with either the **a** or α allele clusters of pathogenic species. This allele-independent placement is thus more consistent with an independent *P/R–HD* fusion in *Cryptococcus* sp. 3. These results then guided our subsequent synteny and karyotype analyses aimed at determining the structural basis of the linkage.

To improve clarity and conciseness, we revised this section substantially and moved the detailed centromere-loss analyses to S2 Text, in line with the reviewers' advice to shorten certain parts of the main text. We also rephrased the transition following the gene phylogeny results to read in **lines 436-438**:

"Having established from gene phylogenies that the MAT fusion in Cryptococcus sp. 3 likely arose independently, we next examined chromosome-scale structural changes underlying the P/R-HD linkage by reconstructing synteny blocks with SynChro [105]."

LINE 535: I recommend clarifying that it's a reciprocal translocation.

Response: Amended as suggested.

LINE 764: Would recommend not using the name "gypsy" for Ty3.

Response: Amended as suggested. The sentence in **lines 690-692** now reads:

"This model had support from several observations: (i) Cryptococcus centromeres are enriched with long-terminal-repeat retrotransposons (LTRs) from the Ty3 and Ty1 superfamilies, which are shared among different centromeres and may facilitate recombination [23, 47, 49, 129, 130];…"

LINE 891: Sharing stress?

Response: Good catch! Rephrased to:

"*High-molecular-weight (HMW) DNA was extracted with a cetyltrimethylammonium bromide (CTAB) extraction as previously described, minimizing DNA shearing during sample preparation".*

LINE 937: What repeat libraries were used?

Response: We thank the reviewer for noting this omission. We have now specified the repeat-masking libraries in the Methods section (**lines 848-851**), which now reads:

Gene models were predicted using either BRAKER2 v2.1.5 [147] or Funannotate v1.8.9 (https://github.com/nextgenusfs/funannotate), as previously described [20, 23, 49]. Prior to gene prediction, assemblies were masked with RepeatMasker v4.1.4 in sensitive mode, applying a custom library that combined Cryptococcus–Kwoniella repeat families generated with RepeatModeler2 v2.0.4 [49], centromere-associated transposable elements, and curated Cryptococcus elements compiled from published sources [148] and represented in Dfam.
